# Precision cancer classification using liquid biopsy and advanced machine learning techniques

**DOI:** 10.1038/s41598-024-56419-1

**Published:** 2024-03-10

**Authors:** Amr Eledkawy, Taher Hamza, Sara El-Metwally

**Affiliations:** 1 Department of Computer Science, Faculty of Computers and Information, Mansoura University, P.O. Box: 35516, Mansoura, Egypt; 2 Biomedical Informatics Department, Faculty of Computer Science and Engineering, New Mansoura University, Gamasa, 35712 Egypt

**Keywords:** Computational biology and bioinformatics, Biomedical engineering

## Abstract

Cancer presents a significant global health burden, resulting in millions of annual deaths. Timely detection is critical for improving survival rates, offering a crucial window for timely medical interventions. Liquid biopsy, analyzing genetic variations, and mutations in circulating cell-free, circulating tumor DNA (cfDNA/ctDNA) or molecular biomarkers, has emerged as a tool for early detection. This study focuses on cancer detection using mutations in plasma cfDNA/ctDNA and protein biomarker concentrations. The proposed system initially calculates the correlation coefficient to identify correlated features, while mutual information assesses each feature's relevance to the target variable, eliminating redundant features to improve efficiency. The eXtrem Gradient Boosting (XGBoost) feature importance method iteratively selects the top ten features, resulting in a 60% dataset dimensionality reduction. The Light Gradient Boosting Machine (LGBM) model is employed for classification, optimizing its performance through a random search for hyper-parameters. Final predictions are obtained by ensembling LGBM models from tenfold cross-validation, weighted by their respective balanced accuracy, and averaged to get final predictions. Applying this methodology, the proposed system achieves 99.45% accuracy and 99.95% AUC for detecting the presence of cancer while achieving 93.94% accuracy and 97.81% AUC for cancer-type classification. Our methodology leads to enhanced healthcare outcomes for cancer patients.

## Introduction

Cancer is widespread globally, resulting in millions of deaths yearly, making it one of the leading causes of mortality worldwide, with approximately 10 million deaths annually^1,2^. Unfortunately, even in developed countries, cancer-related deaths are expected to rise^3^. Genetic alterations are a known cause of cancer, leading to uncontrolled cell growth and evolutionary changes^4–6^. Without systemic therapy, localized cancers can often be successfully treated through surgery alone^1^. However, once distant metastasis occurs, surgical excision is rarely curative. Detecting cancers before they metastasize to distant sites is a major focus in cancer research. Detecting cancer at an early stage allows for early medical interventions, reducing patient mortality rates^7^.

Early cancer detection is now accessible through various methods^8^. A widely adopted method for early cancer identification now involves liquid biopsy, a non-invasive diagnostic approach that detects mutations and genetic variations in circulating cell-free DNA (cfDNA), circulating tumor DNA (ctDNA), and molecular biomarkers^9^. In particular, protein biomarkers, sourced from blood plasma with minimal invasiveness to patients^10^, offer specificity and sensitivity for clinical cancer detection, management, and monitoring^11^. Numerous biomarkers have been introduced for early and late-stage cancer detection across different cancer types^12^. Employing a multi-analytic test approach, which evaluates mutations in cfDNA, ctDNA, and various protein biomarkers, has the potential to augment early cancer detection through the application of machine learning methodologies and tools^13^.

In this study, we employ the term 'cancer detection' to denote the process of identifying the presence of cancer in an individual. Concurrently, we utilize 'cancer classification' to indicate the process of determining the cancer type. This paper focuses on integrating protein biomarker concentrations and mutations in cfDNA/ctDNA from both cancer patients and healthy controls to enhance early cancer detection and classification. The proposed methodology employs a rigorous approach, involving several key steps. The approach begins by using the correlation coefficient to identify correlated features and Mutual Information (MI) calculations to evaluate feature relevance to the target variable. This process is further refined through iterative application of eXtreme Gradient Boosting (XGBoost) feature importance. The Light Gradient Boosting Machine (LGBM) classifier, with hyperparameters optimized through random search, is chosen for cancer detection and classification.

The contributions of this paper can be summarized in the following points:

- Non-invasive liquid biopsy: leveraging protein biomarker concentrations and mutations in plasma cfDNA/ctDNA, the system offers a solution for the early detection and classification of seven types of cancer, including colorectal, breast, upper gastrointestinal (GI), lung, pancreas, ovarian, and liver cancer. This non-invasive approach enhances the patient outcomes and enables timely intervention and treatment.
- Two-step feature reduction: the methodology starts by utilizing the correlation coefficient to identify correlated features in the dataset. Mutual information is then calculated to assess the relevance of each pair of correlated features to the target variable, leading to the removal of redundant features and improved efficiency.
- XGBoost feature importance: the study employs XGBoost feature importance to iteratively select the top 10 most critical features, significantly reducing dataset dimensionality by 60% and improving the model interpretability.
- Ensemble modeling: the LGBM classifier is used, and optimal model performance is achieved using a random search for hyperparameters. An ensemble of LGBM models is created from tenfold cross-validation. The models' predictions are weighted and averaged, contributing to the final predictions.
- Promising results: the methodology achieves an accuracy of 99.45% and a 99.95% AUC for cancer detection while achieving 93.94% accuracy and 97.81% AUC for cancer classification.

The paper is structured as follows: the “Related work” presents an overview of prior work utilizing liquid biopsy and protein biomarkers. The “Materials and methods” provides insights into the dataset used and the methodology employed in our proposed system. The “Experimental results” explains the conducted experiments using the proposed approach and the corresponding results. The “Discussion” includes a discussion of the proposed methodology. Finally, the “Conclusion” concludes the paper and provides insights into future research directions.

## Related work

cfDNA-based liquid biopsy holds promise as a clinical application for early cancer detection^14^. Utilizing blood samples to examine cfDNA offers a non-invasive and convenient medical test for detecting cancer in patients^15^. Researchers are actively exploring somatic variations in circulating cfDNA as a means of early cancer detection across a range of cancer types^13,15,16^, including gastric^17^, colorectal^18^, lung^19^, breast^20^, early-stage lung^21^, and late-stage human malignancies^22^. The ABEMUS^23^ is geared towards identifying somatic single-nucleotide variants in cfDNA for both cancer detection and monitoring recurrent cancer growth using sequencing data from cancer patients^24^.

Machine learning algorithms are being explored for cancer detection using liquid biopsy data. Examples include network-based multi-task learning models^25^, deep learning^26^, and conjunctive Bayesian networks^27^. Current research endeavors in early cancer detection are centered around examining mutations in cfDNA alongside a range of protein biomarkers utilizing blood test analytics, with notable methods like CancerA1DE^28^ CancerSEEK^13^, CancerEMC^9^, and DEcancer^12^. Overall, the growing body of research in cfDNA-based liquid biopsy and machine learning algorithms for early cancer detection demonstrates the potential for transforming cancer diagnosis and management, paving the way for improved patient outcomes and healthcare advancements. In the rest of this section, we explain all related work that utilized the dataset published by Cohen et al.^13^ that we used in the proposed system.

In their study, Cohen et al.^13^ conducted a blood test called CancerSEEK, which comprised protein biomarker concentrations and cfDNA/ctDNA mutation data. They used a straightforward optimization technique to classify cancer patients from healthy individuals. They first removed any protein that showed higher median values in normal samples than cancer samples, identified using a Mann–Whitney-Wilcoxon test. The filtration process resulted in 26 protein biomarkers for further assessment. A forward selection approach was then applied, considering the relevance of each feature based on the decrease in accuracy of logistic regression when that protein was removed from the remaining 26 protein features. The selected 9 features achieved 77.71% accuracy for cancer detection based on a logistic regression classifier. They used data for 626 cancer patients and all 39 protein biomarkers with the omega score plus gender for classifying seven cancer types after grouping patients with esophageal and gastric cancers together, where they achieved 62.32% using a random forest classifier. All experiments are done using a tenfold cross-validation technique.

Wong et al.^28^ proposed a system based on Aggregating One-Dependence Estimators (A1DE) called CancerA1DE. CancerA1DE utilizes A1DE^29^, a semi-naïve Bayesian machine learning technique that weakens the attribute independence assumption on the naïve Bayes classier and aggregates all one-dependence classifiers. The authors proposed a cancer detection system based on the same dataset containing protein biomarker concentrations and cfDNA/ctDNA mutations^13^. By selecting eight protein biomarkers along with the omega score, CancerA1DE achieved an accuracy of 96.64% in detecting cancer cases. They were able to classify seven types of cancer using 39 protein biomarkers, in addition to the omega score and gender. They used tenfold cross-validation as a testing technique in their experiments.

Rahaman et al.^9^ proposed a bagging Ensemble Meta Classifier called CancerEMC using Average One-Dependent Estimators (AODE) for cancer classification. They proposed a system for cancer detection that used clinical blood test data from^13^. Random Forest Feature Selection (RFFS) techniques were employed to pick the optimum protein biomarker attributes. They achieved 99.17% accuracy for cancer detection using 15 biomarkers features from RFFS and omega score plus age, ethnicity, and sex. Using 626 patient data, they achieved 74.12% accuracy for classifying the seven cancer types with 19 biomarker features from RFFS plus omega score and sex on the data before using the Synthetic Minority Over-sampling Technique (SMOTE). After SMOTE, the accuracy of cancer classification has been increased to 91.5%. When doing cancer localization on the 1817 individuals for cancer type classification, they achieved 83.49% accuracy before SMOTE and 95.98% after SMOTE. When doing cancer localization using 1005 cancer patients, they achieved 74.22% accuracy before SMOTE and 93.98% after SMOTE. All results are calculated using a tenfold cross-validation technique.

Halner et al.^12^ used clinical blood test data from^13^ to propose a DEcancer framework for cancer detection. The framework begins by splitting the data into 80% training and 20% testing sets. The training set is further divided using a 200-fold Monte Carlo cross-validation scheme. Multiple data augmentation techniques are applied to enhance the training data. Feature selection and hyper-parameter optimization of the classifier model are performed based on their performance on the validation folds. To evaluate the classification model, an independent t-test is employed to compare its performance with the best-performing feature set to that of the classifier using the smallest subset of variables. The goal is to ensure that the model's performance with the smaller subset is not statistically significantly lower than that of the best feature set. Once the best classifier models and feature set are determined, re-training is performed on the entire training set without using the cross-validation scheme. Finally, the selected classifier models and feature sets are tested on the independent test set to obtain the final results. Using the full 39 biomarker feature in addition to omega score, age, sex, and ethnicity, the framework achieved 99.91% AUC while using only 28 biomarkers, the framework achieved 99.63% AUC for cancer detection. The framework achieved an average of 91.88% AUC for cancer type classification vs. other cancers and an average of 94.13% AUC for cancer type classification vs. other cancers and normal individuals.

The summary of the related work that used the clinical blood test data from^13^ for cancer detection and cancer classification is presented in Tables 1 and 2 respectively.

**Table 1:** Comparison of cancer detection models using Cohen et al. dataset.

Study	Method	# features	Accuracy (%)	AUC (%)
Cohen et al., 2018	CancerSEEK	9	77.71	93
Wong et al., 2019	CancerA1DE	9	96.64	99.1
Rahaman et al., 2021	CancerEMC	18	99.17	99.9
Halner et al., 2023	DEcancer	43	N/A	99.91
		28		99.63

**Table 2:** Comparison of cancer classification models using Cohen et al. dataset.

Study	Method	#Samples	#Features	Accuracy (%)	AUC (%)
Cohen et al., 2018	CancerSEEK	626	41	62.32	91
Wong et al., 2019	CancerA1DE	626	41	69.64	92.1
Rahaman et al., 2021	CancerEMC	626	21	74.12	93.8
		1005	41	74.29	N/A
		1817	43	83.49	98
Halner et al., 2023	DEcancer	1005	43	N/A	91.88
		1817	43		94.13

However, it is important to mention that the CancerSEEK^13^ method has a limitation where its cancer detection component relies on logistic regression, which assumes a linear relationship between different markers. This assumption may not always hold in practice, reducing the method's suitability in particular situations. Similarly, the CancerA1DE^28^ method employs generative learning for cancer classification, which may not be entirely realistic. Generative learning techniques learn from the distribution of data, and this distribution can vary among different datasets, potentially limiting the generalization of the proposed approach. The CancerEMC^9^ approach also utilizes ensemble methods, specifically random forests and bagging classifiers. While these modeling techniques can be effective, they are often challenging to interpret, making it difficult to gain insights into the underlying mechanisms of the classification process. Lastly, the DEcancer^12^ framework is not directly applicable in some cases as it lacks clear information about the set of classifiers and feature selectors used. Furthermore, the study does not specify which classifiers and feature selectors they propose as the best, which can hinder the reproducibility and comparability of the results.

Although the previously mentioned methods offer valuable advantages, it is crucial to be aware of their limitations. Addressing these limitations may pave the way for more accurate and interpretable cancer detection methods in the future. To tackle these constraints, we have introduced a detailed framework, which will be explained in detail in the following section.

## Materials and methods

**Figure 1:**
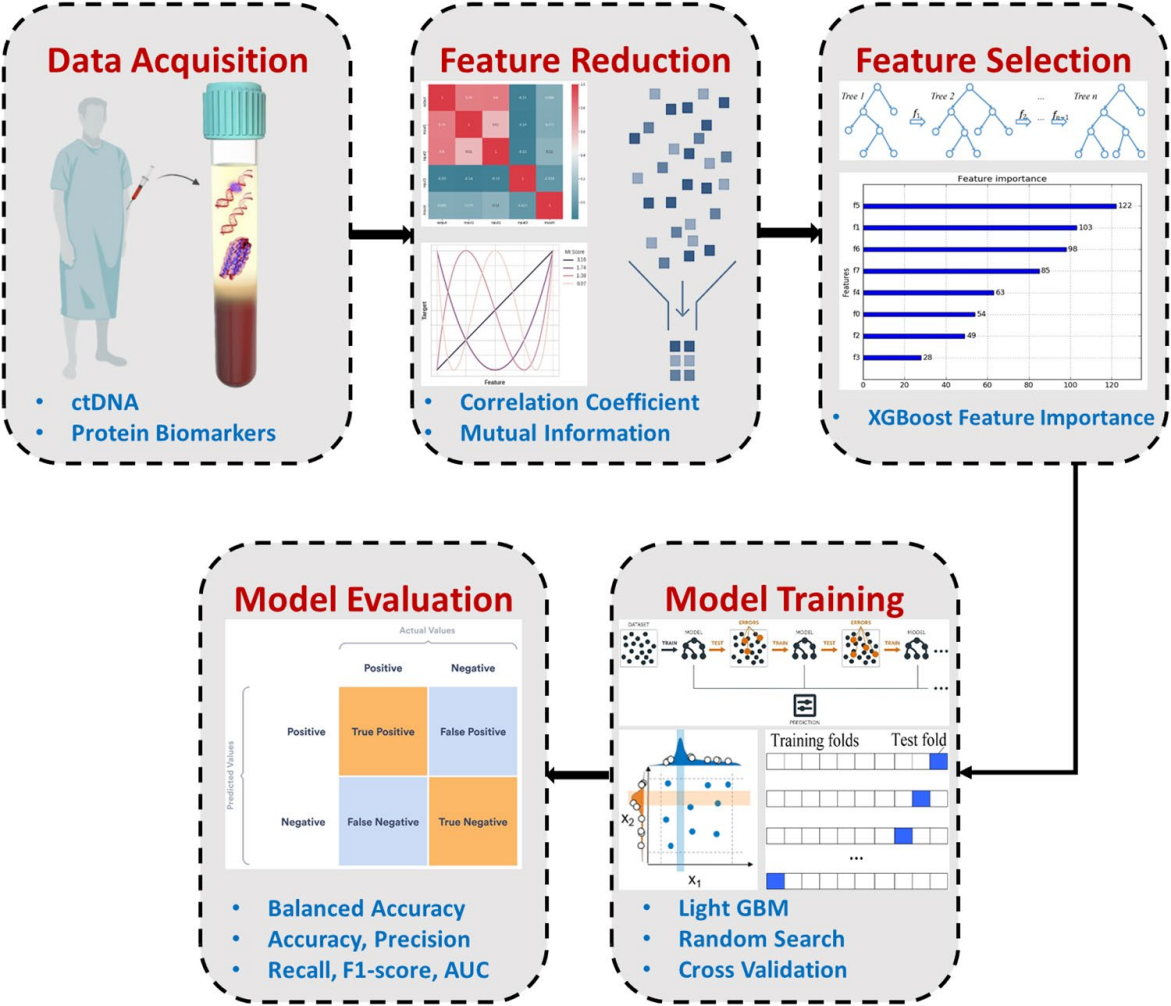
Precision cancer classification pipeline using liquid biopsy and advanced machine learning techniques.

The main goal of this research is to enhance early cancer detection by combining protein biomarkers with cfDNA/ctDNA mutations. Our proposed methodology employs a systematic and robust approach to cancer classification, encompassing several key steps: data acquisition, feature reduction, feature selection, model training, and evaluation (see Fig. 1).

In the initial phase, data is collected from individuals through blood samples, which are then used to extract both protein biomarkers and ctDNA mutations, along with relevant clinical information. Subsequently, we employ feature reduction techniques to eliminate redundant features. This reduced feature set is subjected to a feature selection process facilitated by the XGBoost classifier. The selected features are integrated into a lower-dimensional dataset, the input for training an LGBM classifier. The optimized LGBM model undergoes training and evaluation, during which various performance metrics are computed. These stages are discussed in detail in the following subsections.

### Data acquisition

**Figure 2:**
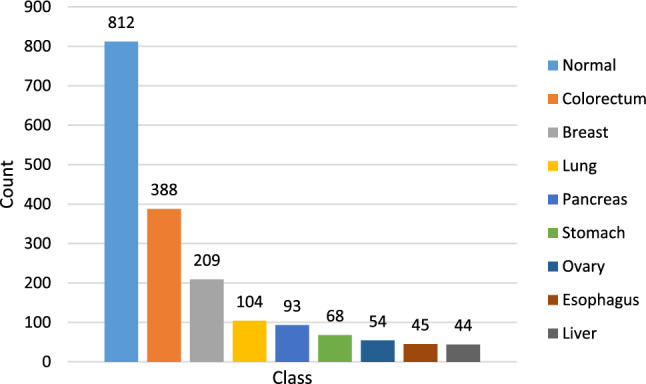
Dataset classes count.

In this study, we utilized a multi-analytic blood test dataset containing mutations of cfDNA/ctDNA and protein biomarker levels in blood plasma samples, along with clinical characteristics, from Cohen et al.^13^. There are 1817 patients' blood test sample data, including 1005 cancer patients diagnosed at an average age of 63. The median age at cancer diagnosis was 64, ranging from 22 to 93 years. These cancers are from various organs, including breast, lung, colorectum, liver, ovary, stomach, pancreas, and esophagus. The eight cancer types were chosen because no common clinical blood-based tests are available for early detection. Additionally, the dataset includes 812 healthy control individuals with an average age of 49. The healthy control group's median age was 55, ranging from 17 to 88 years. These individuals had no history of cancer, high-grade dysplasia, autoimmune disease, or chronic kidney disease. All patients included in the dataset did not receive neo-adjuvant chemotherapy before blood sample collection, and none of them had evident distant metastasis at the time of study entry. The demographic characteristics of the dataset are shown in Table 3. The class counts in the dataset are shown in Fig. 2. Esophageal and gastric cancers are grouped to form a class called upper GI as suggested by Cohen et al.^13^.

**Table 3:** Cohen et al. dataset demographic characteristics.

	Normal (812)	Cancer (1005)
Age (years) (mean, median)	17–88 (49, 55)	22–93 (63, 64)
Sex		
Male	434 (23.9%)	462 (25.4%)
Female	378 (20.8%)	543 (29.9%)
Race		
Asian	22 (1.2%)	301 (16.6%)
Black	154 (8.48%)	14 (0.8%)
Caucasian	332 (18.27%)	675 (37.1%)
Hispanic	76 (4.2%)	1 (0.05%)
Other	228 (12.5%)	14 (0.8%)

The dataset contains 39 protein biomarker concentrations in plasma samples and an omega score calculated from the same patient's detected mutations in cfDNA samples. The 39 protein biomarker features are described in Supplementary File Table S1. The Omega score feature represents the cfDNA mutation score, calculated based on the Mutant Allele Frequency (MAF) observed in four wells of Unique Identifier sequences (UIDs) of cfDNA present in plasma samples. The calculation of the Omega score is done using Eq. (1).

1 $$\Omega =\sum_{i=1}^{w}{w}_{i}\times \mathit{ln}\frac{{p}_{i}^{C}}{{p}_{i}^{N}}$$

In Eq. (1), $w$ represents the number of wells in the cfDNA sample. ${w}_{i}$ is calculated as the ratio of ${UIDs}_{i}$ (the number of Unique Identifiers in the *i*th well of plasma sample $s$) to $UIDs$ (the total number of Unique Identifiers). ${p}_{i}^{C}$ denotes the p-value of cancer samples $C$ in the MAF distribution of the *i*th well, while ${p}_{i}^{N}$ represents the p-value of normal samples $N$ in the MAF distribution of the same well.

Clinical characteristics such as age, sex, race (ethnicity), and histopathology are also included in the dataset. However, the histopathology feature, which describes the microscopical characteristics of cancer cells/tissues to identify different cancer types, is not used as an input feature for cancer detection in this study. The 'sex' feature plays a significant role in some cancer types, such as breast and ovarian cancer, as it shows discriminative characteristics in female patients. Additionally, the ethnicity feature represents an individual's genetic invariant and physical traits, which can also impact binary cancer detection. Overall, this dataset provides a comprehensive set of features and clinical characteristics that can aid in early cancer detection and contribute to the development of effective diagnostic methods for various cancer types.

### Feature reduction

The Pearson correlation coefficient (Eq. 2)^30^ is a statistical measure employed to identify correlated variables represented by features. This coefficient, denoted as $r$, is calculated using the concentrations of two compounds, $X$ and $Y$, across $N$ data points. $r$ can vary between + 1 and −1, with positive values indicating a positive linear correlation between $X$ and $Y$. A '0' signifies no linear correlation, while negative values suggest a negative linear correlation. The sign of $r$ reflects the direction of the relationship, while the magnitude of it signifies the strength of the linear relationship.

2 $$r=\frac{N\sum XY-(\sum X\sum Y)}{\sqrt{\left[N\sum {X}^{2}-{\left(\sum X\right)}^{2}\right]\left[N\sum {Y}^{2}-{\left(\sum Y\right)}^{2}\right]}}$$

For each pair of correlated variables $X$ and $Y$, we calculate the MI of each feature with the target class. MI is calculated using Eq. (3), where $I(X;Y)$ represents the MI for $X$ and $Y$, $H(X)$ is the entropy of $X$, and $H\left(X|Y\right)$ is the conditional entropy of $X$ given $Y$^31^.

3 $$I\left(X;Y\right)=H\left(X\right)-H\left(X|Y\right)$$

Entropy measures the information content in a random variable. It represents the uncertainty in the variable's possible outcomes. Mutual information, on the other hand, measures the dependency between two random variables. Mutual information plays a crucial role in reducing the entropy of the variables involved. When two variables are independent (i.e., have no relationship), their mutual information is zero, indicating no reduction in entropy. Conversely, higher mutual information between the variables implies a greater reduction in entropy, signifying a stronger relationship or dependency between them. Thus, mutual information helps in reducing the uncertainty (entropy) in the joint distribution of two random variables by providing insights into their dependency and relationship.

As a result, we exclude the feature with the lowest MI with the target class and retain the one with the highest MI with the target class. The output of this phase is the reduced set of features, which includes only uncorrelated ones.

### Feature selection

XGBoost employs an iterative approach that constructs new trees while focusing on minimizing a defined loss function, thus enhancing predictive accuracy. The formulation of XGBoost can be expressed as shown in Eq. (4):

4 $${\hat{y}}_{i}=\sum_{k=1}^{n}{f}_{k}({x}_{i}),{f}_{k}\in F$$

Here,$F$ represents the space of trees, ${f}_{k}$ corresponds to an individual tree, ${f}_{k}({x}_{i})$ denotes the outcome of tree $k$, and ${\hat{y}}_{i}$ indicates the predicted value for the *i*th instance, ${x}_{i}$. The aim is to minimize the loss function, as expressed in Eq. (5):

5 $$obj\left(\theta \right)=\sum_{i}l\left({\hat{y}}_{i},{y}_{i}\right)+\sum_{k}\Omega \left({f}_{k}\right)$$

In Eq. (5), $l$ represents the loss function, ${\hat{y}}_{i}$ indicates the prediction, and ${y}_{i}$ is the target value. Furthermore, $\Omega$ introduces a penalty that considers the number of leaves within the model and their associated scores. It seeks to strike a balance between model complexity and predictive accuracy. Adjusting the value of $\Omega$ enables the algorithm to regulate the level of model regularization, thereby mitigating overfitting and enhancing generalization to unseen data. A higher $\Omega$ value encourages a simpler model with fewer leaves, while a lower $\Omega$ value permits a more complex model with a greater number of leaves, thus influencing overall model performance.

To identify the most important features, we employ XGBoost in the feature selection process. The entire dataset is initially used, and the top 10 features are determined. This process is iteratively repeated five times. The resulting sets of features from each iteration are combined, and any duplicate features are eliminated.

### Model training

The LGBM^32^ uses the histogram algorithm^33^ that converts each column of eigenvalues into a histogram, generating $k$ bins based on integer intervals. Then, it places the eigenvalues into bins corresponding to their intervals. This ensures that the number of bins is less than the number of features, reducing memory usage and computational complexity. The LGBM process involves several phases. Initially, $n$ decision trees are initialized, and the weight of the training set is set to $\frac{1}{n}$. In the second phase, weak classifiers are trained. Subsequently, each classifier's weight is considered, and updates are made accordingly. Finally, the final classifier is obtained as shown in Eq. (6):

6 $${H}_{T}\left(X\right)=\sum_{t}h\left(x,{\theta }_{t}\right)$$

Here, $h\left(x,{\theta }_{t}\right)$ represents the prediction result after training a tree, and ${\theta }_{t}$ is the output parameter of the decision tree model for input $x$ in tree $t$. We employ a random search technique for hyperparameter optimization in the LGBM model. This training process includes performing tenfold cross-validation utilizing the optimized LGBM model, which yields ten trained models with their associated balanced accuracy.

### Model evaluation

Several evaluation metrics are employed, including accuracy, precision, recall, F1-score, and others. These metrics are defined in terms of True Positives (TP), False Positives (FP), False Negatives (FN), and True Negatives (TN). TP represents correctly classified cancer patients, while FP denotes wrongly classified cancer patients. TN indicates correctly classified normal individuals, and FN represents wrongly classified normal individuals.

Accuracy, as a measure of the model's correctness, is calculated as the ratio of correct classifications to all classifications (Eq. 7). Precision, on the other hand, quantifies the fraction of true positives among the retrieved positive samples (Eq. 8), with low precision indicating a large number of false positives. Recall, also known as True Positive Rate (TPR) or sensitivity, measures the fraction of true positives retrieved over the total number of actual positive samples (Eq. 9), with a high recall indicating a small number of misclassified positive samples. The F1-score, calculated using Eq. (10), represents the harmonic mean between precision and recall, offering a balanced measure of the model's performance^34^.

7 $$accuracy=\frac{TP+TN}{TP+FP+FN+TN}$$

8 $$precision=\frac{TP}{TP+FP}$$

9 $$recall=\frac{TP}{TP+FN}$$

10 $$F1-score=\frac{2*precision*recall}{precision+recall}$$

Additionally, the AUC is utilized as an evaluation measure, representing the area under the Receiver Operating Characteristics (ROC) curve. The ROC curve plots the False Positive Rate (FPR) on the x-axis and the TPR on the y-axis. FPR is calculated as the percentage of incorrectly predicted negative cases (Eq. 11), and the AUC result ranges from 0 to 1, with a higher value signifying better model performance in correctly classifying positive and negative instances. Balanced accuracy is the average of TPR and True Negative Rate (TNR). TNR or specificity measures the fraction of negatives correctly classified among all negatives (Eq. 12). Balanced accuracy metric is particularly useful with imbalanced data, and it can be calculated using Eq. (13)^35^.

11 $$FPR=\frac{FP}{FP+TN}$$

12 $$specificity=\frac{TN}{TN+FP}$$

13 $$balanced \,accuracy=\frac{recall+specificity}{2}$$

## Experimental results

In this section, we present the experimental results obtained through our proposed system methodology. Initially, the system is provided with a dataset comprising 39 cfDNA/ctDNA biomarker features, along with three clinical features encompassing sex, age, and race. Additionally, the dataset includes the omega score feature, resulting in a total of 43 features. These features serve as inputs for various phases within our methodology, with each phase producing results that are subsequently utilized in the subsequent stages.

**Figure 3:**
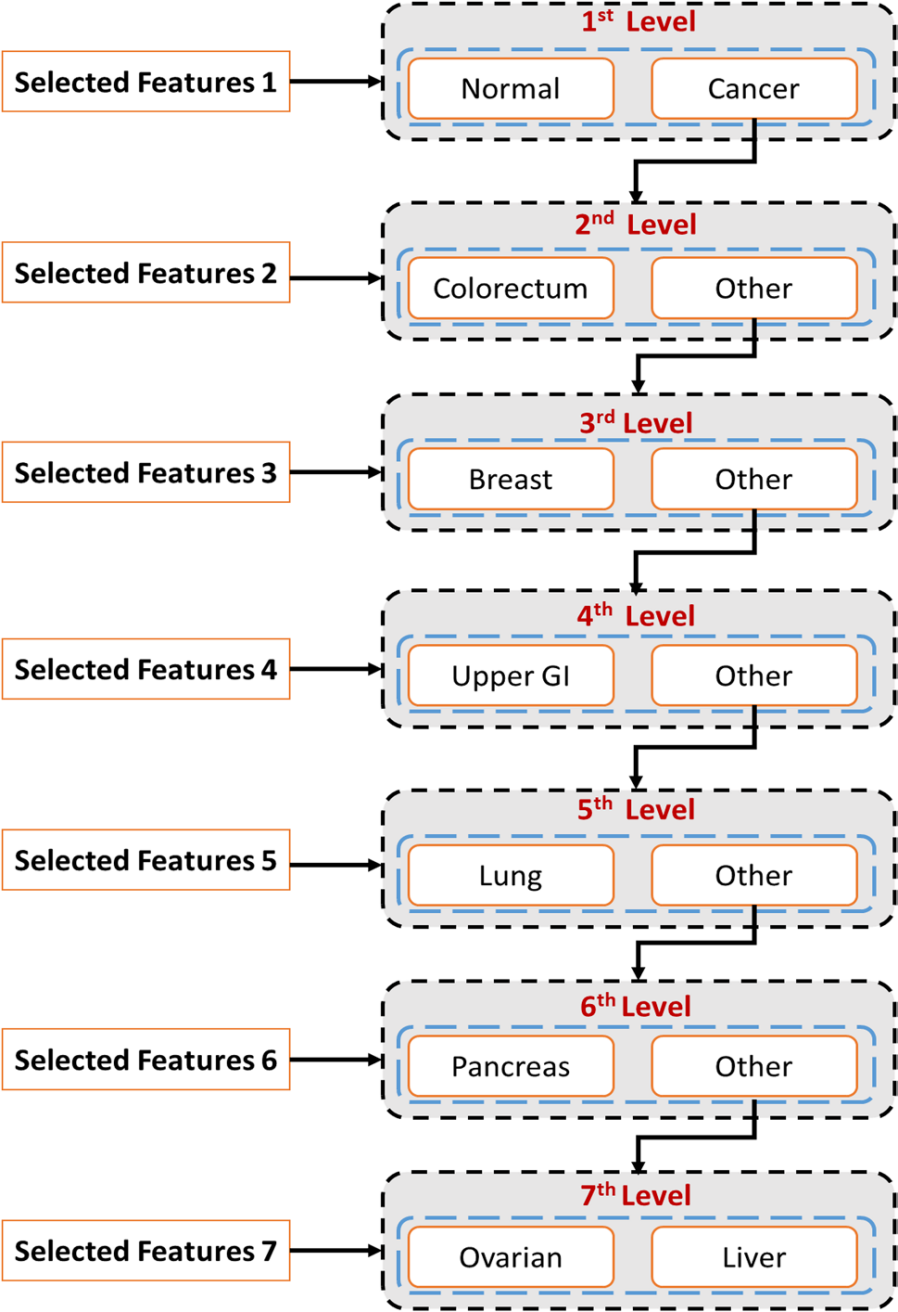
Multi-level cancer classification process.

The experiments for cancer diagnosis leverage a multi-stage binary classification approach. This framework progressively refines the data through iterative levels, enabling more precise identification of various cancer types. The initial stage employs the entire dataset for a preliminary binary classification task, distinguishing between normal and cancerous samples. This initial classification lays the foundation for subsequent stages. Following cancer detection, the 'normal' instances are removed. The remaining data then informs the creation of a new dataset with binary labels: 'target cancer' or 'other cancers'. This stage focuses on differentiating patients with the specific cancer of interest (e.g., colorectal) from those with other malignancies. This iterative process continues, each time removing the identified 'target cancer' from the data and creating a new binary classification dataset. Subsequent stages address other cancer types similarly, progressively narrowing down the focus. Through this stage-wise refinement, seven distinct datasets are generated, each suitable for binary classification and focusing on a specific cancer type against a background of remaining malignancies. Figure 3 visually illustrates the multi-stage binary classification process employed in our experiments.

To mitigate the risk of overfitting, all datasets are initially partitioned into a 10% test set and a 90% training set. Consequently, the calculations for feature reduction and selection were exclusively performed on the training set rather than the entire cohort. The detailed results of each phase in our proposed methodology are provided in the following subsections.

### Feature reduction

**Figure 4:**
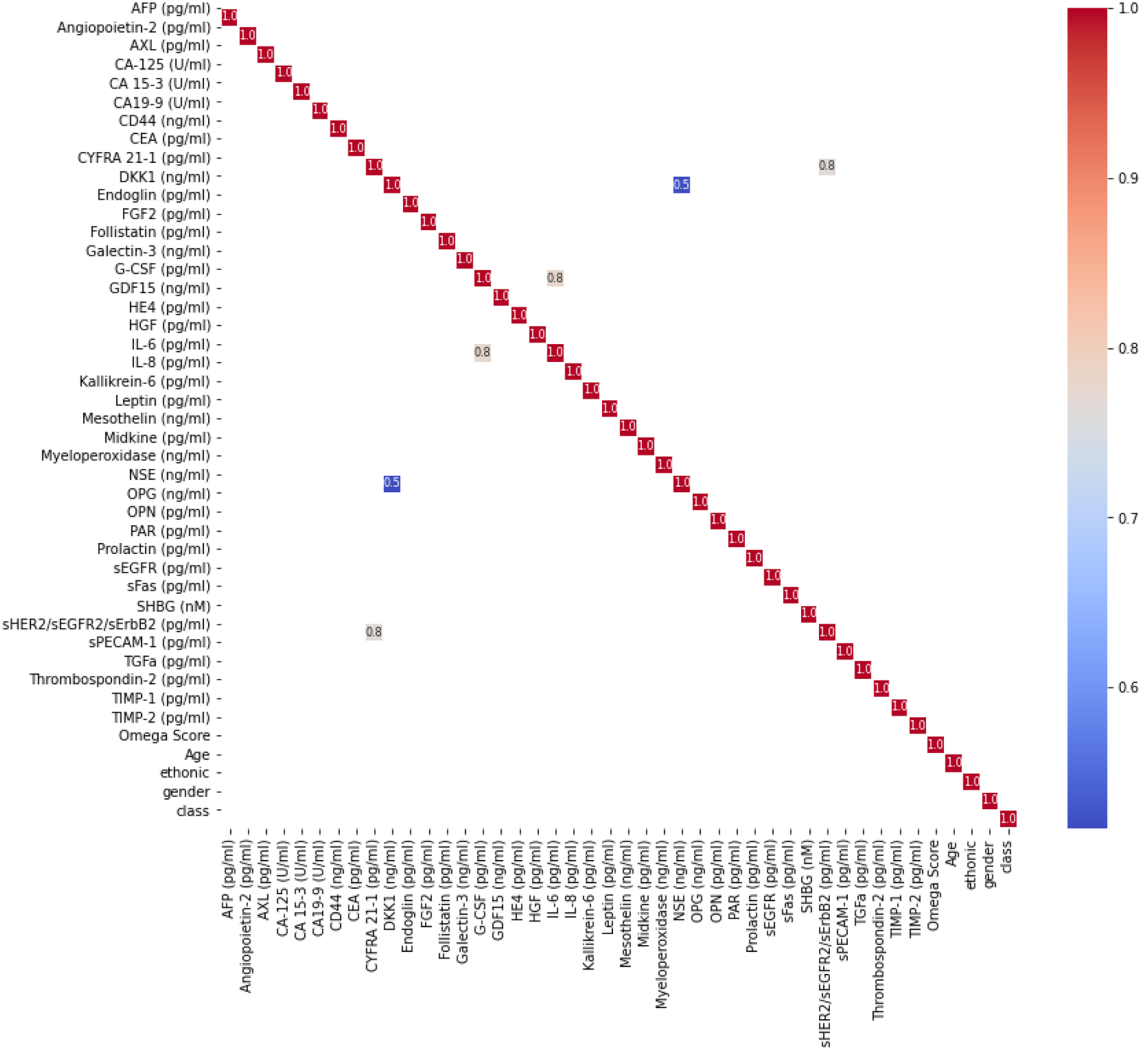
Correlation heatmap of features (*|Correlation|*> 0.5).

For cancer detection, in the feature reduction phase, we consider features that have an absolute correlation value greater than 0.5 as correlated. Based on this condition, we found three pairs of correlated features. Supplementary File Table S2 shows the pairs alongside the |Correlation| value. Figure 4 shows the correlation heatmap between the features showing only the correlation values with |Correlation| greater than 0.5. We proceeded by calculating the MI for the four pairs of correlated features with the target class. The results are summarized in Supplementary File Table S3, which presents the MI values between the three pairs and the target class, indicating which features were retained and which were removed from the dataset.

This feature reduction step led to the removal of three features: G-CSF, sHER2/sEGFR2/sErbB2, and DKK1. Consequently, the dataset was reduced from its original 43 features to 40 features. The same feature reduction procedure continues for the subsequent datasets, with each stage's results detailed in Supplementary File Table S4.

### Feature selection

For cancer detection, the reduced feature set is then iteratively processed by XGBoost over five rounds. Initially, we determine a subset size equal to one-fifth of the reduced set, resulting in 8 features per round ($40/5=8$). Consequently, each round involves a dataset with a progressively decreasing number of features: the first round starts with 40 features, the second with 32, the third with 24, the fourth with 16, and the final round comprises 8 features. During each round, the dataset is utilized to train the XGBoost classifier, where feature importance is computed, and the top ten important features are subsequently identified.

Feature importance is determined by XGBoost through the calculation of gain. For each feature, XGBoost computes gain, which signifies the enhancement in the loss function as a result of dividing the data based on that particular feature. A higher gain value denotes greater feature importance. In our XGBoost setup, we employed the misclassification error rate^36^ as the chosen loss function, which can be quantified using Eq. (14). It's essential to note that gain computation in XGBoost is intimately linked to the optimization process employed during boosting.

14 $$Error=\frac{FP+FN}{TP+FP+FN+TN}$$

**Figure 5:**
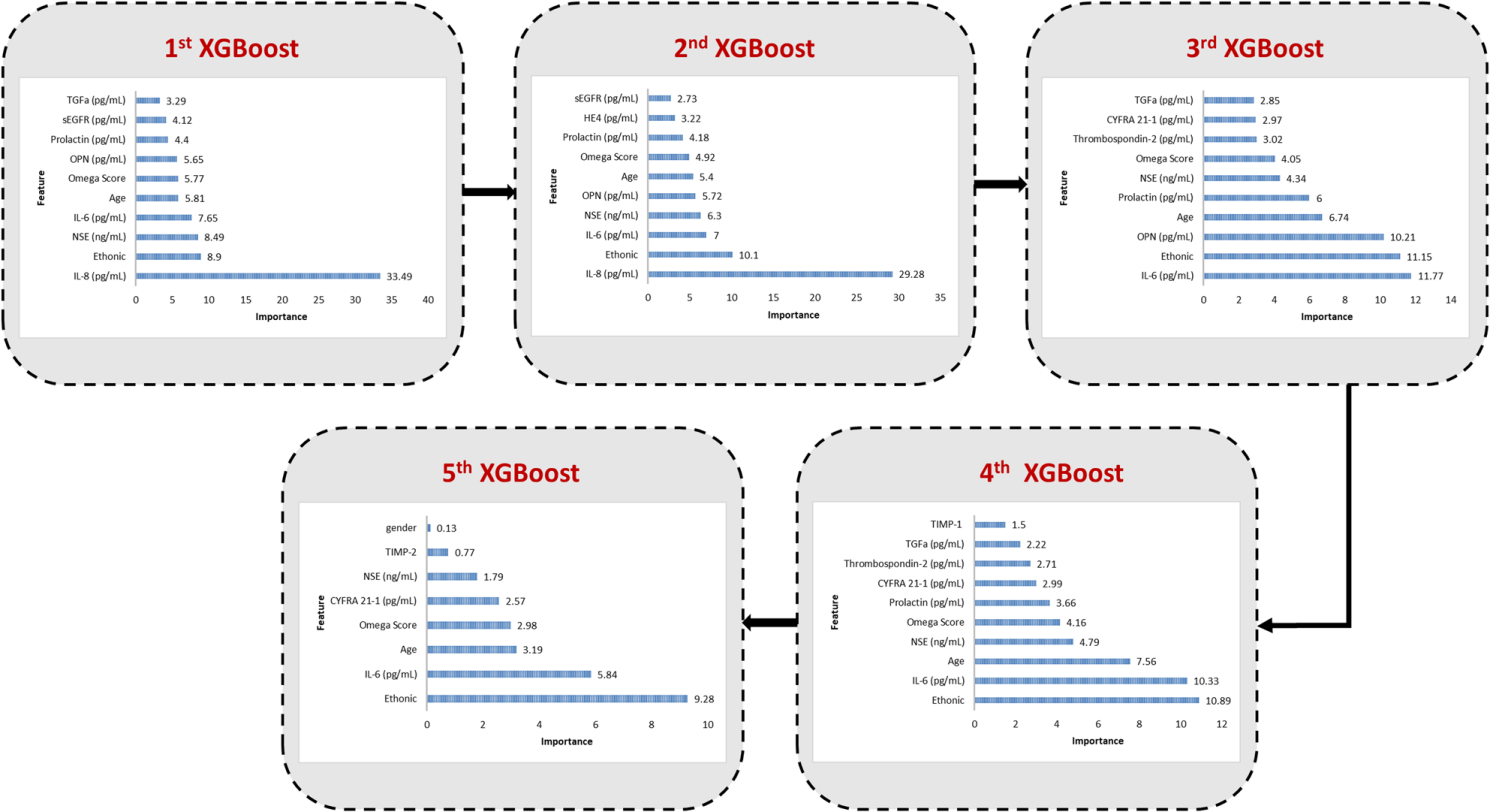
Visualization of feature selection results from XGBoost rounds.

Figure 5 illustrates the outcomes of selecting the top essential features from each of the five XGBoost rounds, respectively. When aggregating the features from all five rounds and removing duplicates, the result is a set of 16 features, which represents the outcome of the feature selection phase. In the initial XGBoost round, the selected features were IL-8, Ethnicity, NSE, IL-6, Age, Omega Score, OPN, Prolactin, sEGFR, and TGFa. Subsequently, in the second XGBoost round, nine features from the previous round were retained, and HE4 was added, bringing the total to 11 features. In the third round, eight features were selected from the previous rounds, and two new features, CYFRA 21-1 and Thrombospondin-2 were included, resulting in a total of 13 features. The fourth XGBoost round retained nine features from the previous rounds and added TIMP-1, resulting in 14 features. However, in the last XGBoost round, only eight features were included due to the limited number available, preventing the selection of a top ten. Six of these features were repeated from the previous cycles, and TIMP-2 and gender features were introduced, resulting in a final count of 16 features.

Consequently, the original dataset has undergone a substantial reduction, decreasing from its initial 43 features to just 16 features, marking a notable 60% reduction in feature count. This reduced feature set will be utilized for model training in the subsequent model training phase. The same feature selection procedure continues for the subsequent datasets, with each stage's results detailed in Supplementary File Table S5.

### Model training and evaluation

For cancer detection, the LGBM model was utilized for model training, and to optimize its hyper-parameters, a random search was conducted. The key hyper-parameters optimized included n_estimators, num_leaves, and learning_rate. The n_estimators parameter determines the number of boosted trees to fit, the num_leaves parameter specifies the maximum number of leaves in a single tree. This parameter significantly influences the model's complexity, with higher values leading to more complex models. Finally, the learning_rate parameter controls the step size shrinkage used in each model update, affecting the magnitude of changes incorporated from each tree's output. During the random search for hyper-parameter optimization, the model's performance was evaluated using the balanced accuracy metric (see Table 4). The best hyper-parameter configuration achieved a balanced accuracy of 98.57%. The same procedure continues for the subsequent datasets, with each stage’s best configuration and balanced accuracy results detailed in Supplementary File Table S6.

**Table 4:** Optimal hyperparameter configuration for LGBM model.

Parameter	Search space	Optimal value
n_estimators	50, 100, 150, 200, 250, 300, 350, 400, 450, 500	150
num_leaves	5, 15, 25, 35, 45, 55, 65, 75, 85, 95, 106, 116, 126, 136, 146, 156, 166, 176, 186, 196, 207, 217, 227, 237, 247, 257, 267, 277, 287, 297, 308, 318, 328, 338, 348, 358, 368, 378, 388, 398, 409, 419, 429, 439, 449, 459, 469, 479, 489, 500	500
learning_rate	Continuous uniform distribution between 0 and 1	0.21

The LGBM model with the best hyper-parameters is then trained on the training set with tenfold cross-validation resulting in ten models, each is validated against one-tenth of the training set and trained on the rest of it. The validation predictions of each model are then used to calculate the balanced accuracy of each model. The average balanced accuracy of the ten trained models is 97.9%. For the remaining datasets, the same procedure follows, and the average balanced accuracies are 83.51%, 85.36%, 81.98%, 83.54%, 89.72%, and 94.16% respectively.

The ten LGBM models are used to make predictions on the test set. The predictions of the ten models are then weighted based on their respective balanced accuracy metrics. Next, we compute the average of these weighted predictions and round it to obtain the final prediction. To validate these predictions, we compare them with the actual test set predictions, enabling the calculation of the confusion matrix to assess the model's predictive capabilities. The confusion matrix of the model after evaluation is shown in Table 5.

**Table 5:** Confusion matrix for cancer detection.

		Predicted	
		Cancer	Normal
Actual	Cancer	100 (TP)	1 (FN)
	Normal	0 (FP)	81 (TN)

**Figure 6:**
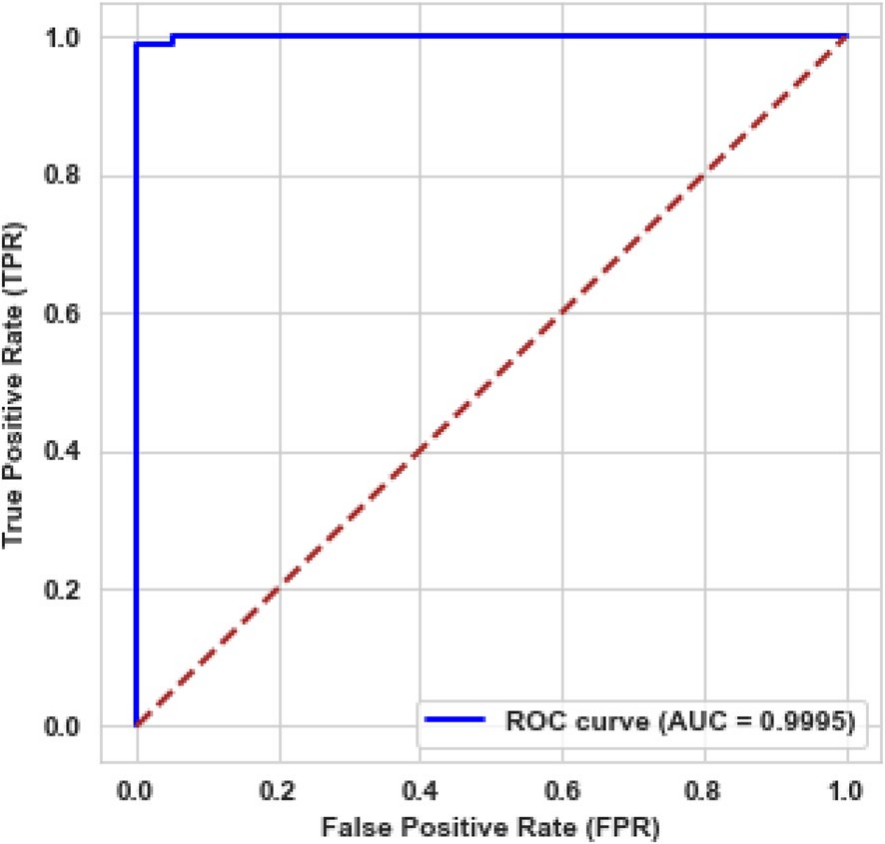
ROC curve for the proposed model of precision cancer classification.

Based on the confusion matrix, the system achieved 99.45% accuracy, 100% precision, 99.01% recall, 99.5% F1-score, and 99.95% AUC. Figure 6 shows the area under the ROC curve. The same procedure continues for the subsequent datasets, with each stage performance results detailed in Supplementary File Table S7. The overall system average performance results across all stages are 93.36% precision, 94.61% recall, 93.85% F1-score, 93.94% accuracy, and 97.81% AUC.

### Benchmarking results

**Figure 7:**
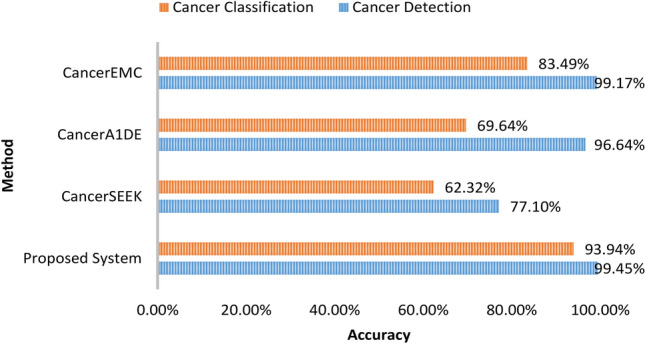
Accuracy comparison: proposed system vs. previous methods.

**Figure 8:**
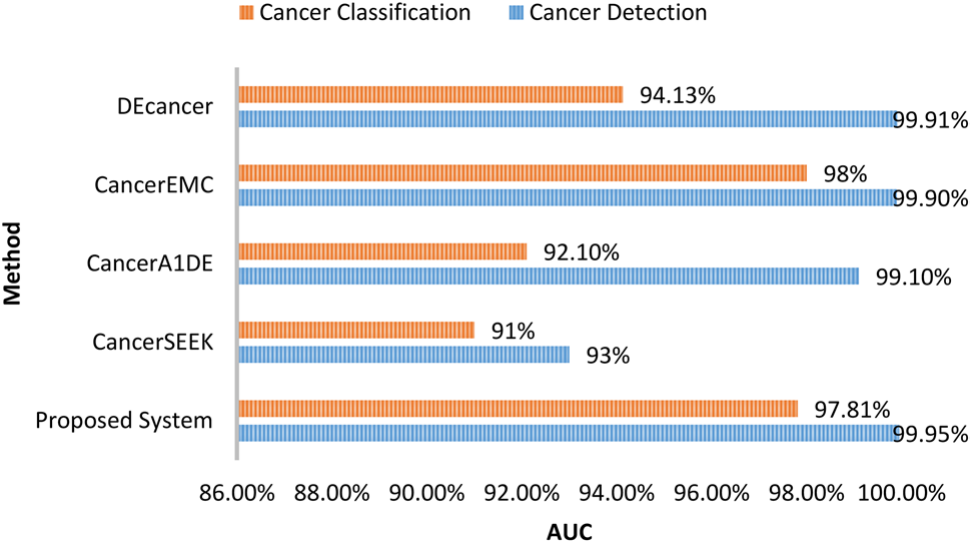
AUC comparison: proposed system vs. previous methods.

We compared the proposed system results for both cancer detection and cancer classification with previous related work. Figure 7 shows a comparison in terms of the accuracy measure between the proposed system, CancerSEEK^13^, CancerA1DE^28^, and CancerEMC^9^. Figure 8 shows a comparison in terms of the AUC measure between the proposed system, CancerSEEK^13^, CancerA1DE^28^, CancerEMC^9^, and DEcancer^12^.

Table 6 shows an AUC and accuracy comparison between the proposed system and other machine learning techniques including Deep Learning^37^, Decision Tree^38^, Naïve Bayes^39^, Support Vector Machine (SVM)^40^, k-Nearest Neighbor (k-NN)^41^, Random Forest^42^, Adaptive Boosting (AdaBoost)^43^, combined Decision Tree and Naïve Bayes (DTNB)^44^, and multi-objective Evolutionary Fuzzy C-means (Evol. Fuzzy C-Means)^9^.

**Table 6:** AUC and accuracy comparison: proposed cancer detection system vs. various machine learning techniques.

Classification approach	Accuracy (%)	AUC (%)
Deep learning	82.05	91.6
Naïve Bayes	77.1	88.9
Decision tree	89.21	91.3
SVM	80.18	81.4
k-NN	76.82	76.2
Random forest	91.9	91.3
AdaBoost	94.55	98.2
DTNB	95.54	99
Evol. fuzzy C-means	76.49	76.7
Proposed system	**99.45**	**99.95**

Significant values are in bold.

The results achieved with our methodology show its effectiveness in cancer classification. The results are indicative of the model's ability to capture the features associated with cancer detection. Our approach not only offers a highly accurate prediction tool but also provides valuable insights into the features relevant to cancer classification, contributing to the field's understanding and potential clinical applications.

Our approach offers some advantages. While the CancerSEEK^13^ method relies on logistic regression with an assumption of linear relationships, our model leverages XGBoost and LGBM techniques, designed to handle non-linear associations more effectively. The generative learning employed by CancerA1DE^28^ may face limitations due to varying data distributions across datasets, whereas our method, based on feature importance and ensemble modeling, does not encounter this issue. Furthermore, our use of LGBM, in comparison to the bagging classifier in CancerEMC^9^, allows for more accurate results. Lastly, our approach provides a more transparent framework compared to DEcancer^12^, enabling system reproduction.

## Discussion

The proposed system provides a non-invasive approach for the detection and classification of seven types of cancer, this enhances patient comfort and facilitates timely intervention and treatment. In this study, we proposed a comprehensive methodology for precision cancer classification that combines several powerful techniques to achieve highly accurate predictions. Using correlation coefficient combined with MI for feature reduction not only streamlined the subsequent analysis but also allowed us to focus on the most relevant features associated with cancer classification. Furthermore, using XGBoost enabled us to improve the interpretability of the model. By considering the most important features, we created a more efficient model without sacrificing predictive performance. The selected features indicate which biomarkers are affecting cancer detection and classification.

To justify the usage of the top 10 features at each XGBoost cycle, we systematically conducted experiments with varying top feature subset sizes, ranging from 5 to 15, to assess the impact on the model's performance. As depicted in Table 7, our findings reveal that the model's accuracy and AUC consistently improve as the number of top feature subset sizes increases. Moreover, as the number of top features surpasses 10, the accuracy tends to plateau or exhibit slight fluctuations, suggesting that the additional information gained from including more features may not significantly contribute to improved accuracy. Therefore, we opted for the model configuration with the highest accuracy while maintaining the least number of features possible. The selected features at the different top feature subset sizes can be found in Supplementary File Table S8.

**Table 7:** Impact of top features subset size on model performance.

#Top features	#Selected features	Accuracy (%)	AUC (%)
5	9	97.25	99.73
8	12	98.9	99.87
10	16	99.45	99.95
12	19	99.45	99.99
15	25	98.9	99.96

Hyper-parameter tuning plays a crucial role in optimizing model performance. Random search allowed us to sample a broader range of hyper-parameter combinations, leading to better model performance. As a result, our methodology produced a model that was less prone to overfitting and had improved generalization capabilities. The tenfold cross-validation ensured a rigorous evaluation of our model and allowed us to obtain more accurate estimates of the model's performance on various subsets of data. Ensembling the LGBM classifiers generated through tenfold cross-validation proved to be a valuable strategy for improving prediction accuracy. This ensemble approach effectively mitigated potential model bias and enhanced the model's performance.

According to^45,46^, six common biomarkers routinely employed in clinical practices (AFP, CA 19–9, CA 125, CEA, Prolactin, and CA 15–3) exhibit an average cost of $2 per test. Other biomarkers in the dataset have a higher average cost of $5.5 per test, likely reflecting their specialized nature. Analysis for each biomarker typically requires an average clinical test time of 2.5 h, based on current laboratory protocols^45,46^.

Table 8 shows a comparison between the proposed cancer detection system and previous ones in terms of clinical cost and time. Cohen et al.^13^, Wong et al.^28^, and classification approaches presented in Table 6 employ a set of nine features for cancer detection, eight of which are biomarkers, with four of these being routine biomarkers. This contributes to their cost and time efficiency in cancer detection, but lower accuracy. Rahman et al.^9^ adopt a panel of 15 biomarkers for cancer detection, of which only one is a routine biomarker, resulting in higher associated costs and testing duration compared to our proposed system. All previous systems employ the complete set of 39 protein biomarkers for cancer-type classification, whereas our proposed system utilizes 35 for this task while achieving better results. Our proposed system, therefore, stands out for its cost and time efficiency without compromising accuracy.

**Table 8:** Comparison of clinical cost and time.

Method	Cancer detection		Cancer classification	
	Cost ($)	Time (h)	Cost ($)	Time (h)
Cohen et al., 2018	30	20	193.5	97.5
Wong et al., 2019	30	20	193.5	97.5
Rahaman et al., 2021	79	37.5	193.5	97.5
Halner et al., 2023	193.5	97.5	193.5	97.5
Proposed system	62.5	30	171.5	87.5

To conduct an independent validation of the proposed cancer detection model, we used the publicly available sample dataset introduced by Hinestrosa et al.^47^, as no other publicly accessible dataset for this purpose was identified to the best of our knowledge. This dataset presents certain limitations, notably the absence of many features from the Cohen et al.^13^ dataset, and the inclusion of patients with bladder cancer which is not a category within the original eight cancer types of the Cohen et al.^13^ dataset.

The Hinestrosa et al.^47^ dataset underwent a preprocessing phase involving the removal of extra features not present in the Cohen et al.^13^ dataset, recalibration of features with differing units, and elimination of features exhibiting noisy distributions. Also, bladder cancer samples were excluded from the dataset. The initial Hinestrosa et al.^47^ sample dataset comprised 42 biomarker readings alongside age and gender features, and a total of 646 samples categorized as 368 healthy controls, 96 with bladder cancer, 94 with pancreatic cancer, and 88 with ovarian cancer. Post-preprocessing, the sample dataset retained 13 biomarker features common to both datasets, in addition to age and gender. Further, after the removal of bladder cancer samples, the dataset was reduced to 550 samples, with 182 instances of cancer.

Employing the proposed pipeline, we trained a cancer detection model on the Cohen et al.^13^ dataset, incorporating solely the common features. The pipeline didn’t remove any feature in the feature reduction step. Subsequently, the pipeline selected 14 features during the feature selection step, encompassing OPN, Age, Prolactin, CA-125, CA19-9, HE4, sFas, FGF2, sPECAM1, TIMP1, Myeloperoxidase, Galectin-3, gender, and Leptin.

When evaluated on the independent Hinestrosa et al.^47^ sample dataset using the selected features, the trained model achieved 82.36% accuracy and 84.83% AUC. This decline can be attributed to the absence of indicative features utilized by the original model on the complete Cohen et al. dataset, which is not present in the more limited Hinestrosa et al.^47^ sample dataset. Despite this, the results remain promising, comparable to the performance of the Cohen et al.^13^ CancerSEEK model and the models presented in Table 7. The confusion matrix results for the Hinestrosa et al.^47^ sample dataset are provided in Supplementary File Table S9.

## Conclusion

Cancer represents a significant global health challenge, leading to millions of deaths annually. Early detection of cancer is of paramount importance, as it has a direct correlation with improved survival rates and outcomes. This study primarily addressed cancer detection by examining protein biomarker concentrations and identified mutations in plasma cfDNA/ctDNA data obtained from both cancer patients and healthy individuals. The proposed methodology begins by using the correlation coefficient to identify correlated features in the dataset, followed by calculating MI to determine the relevance of each feature concerning the target variable. Redundant features are removed, improving efficiency. Further refinement is achieved through XGBoost feature importance, where the top 10 most important features are identified iteratively, significantly reducing dataset dimensionality by 60% and enhancing model interpretability. For the classification task, the LGBM classifier is employed, and optimal model performance is achieved using a random search for hyper-parameters. The methodology achieved 99.45% accuracy and 99.95% AUC for cancer detection along with achieving 93.94% accuracy and 97.81% AUC for cancer type classification.

The future directions of this research can be categorized into three key areas. Firstly, exploring the incorporation of additional types of molecular data, such as RNA or epigenetic markers, holds the potential to increase the sensitivity of cancer detection. Secondly, the utilization of longitudinal data to monitor shifts in protein biomarker concentrations and genetic mutations over time offers a dynamic insight into cancer progression and treatment response. Lastly, delving into the potential applications of our methodology within specific cancer subtypes or in conjunction with other diagnostic modalities may pave the way for precisely targeted therapeutic interventions.

### Supplementary Information

Supplementary Tables.

## Data Availability

Data will be made available on request from the corresponding author S.E.
