# Supplementary material for: Precision cancer classification using liquid biopsy and advanced machine learning techniques
Source: Sci Rep. 2024 Mar 10;14:5841. doi: 10.1038/s41598-024-56419-1 (PMC10925597; doi:10.1038/s41598-024-56419-1)
Supplement: Supplementary file 1 — Supplementary Tables. [file 41598_2024_56419_MOESM1_ESM.docx]

**Table S1:** Protein Biomarker Features Description of Cohen et al. Dataset.

| Input Features | Feature Description |
| --- | --- |
| TGFa (pg/mL) | Circulating Transforming Growth Factor |
| HE4 (pg/mL) | Circulating Human Epididymis Protein 4 |
| sFas (pg/mL) | Circulating soluble Fas Cell Surface Death Receptor |
| Thrombospondin-2 (pg/mL) | Circulating Thrombospondin-2 |
| AFP (pg/mL) | Circulating Alpha Fetoprotein Precursor |
| G-CSF (pg/mL) | Circulating Granulocyte-Colony Stimulating Factor |
| IL-6 (pg/mL) | Circulating Interleukin-6 |
| CA-125 (U/mL) | Circulating Cancer Antigen 125 |
| sHER2/sEGFR2/sErbB2 (pg/mL) | Circulating sHER2/sEGFR2/sErbB2 |
| TIMP-2 (pg/mL) | Circulating Tissue Inhibitor of Metalloproteinases 2 |
| CD44 (ng/mL) | Circulating CD44 |
| CA19-9 (U/mL) | Circulating Cancer Antigen 19-9 |
| IL-8 (pg/mL) | Circulating Interleukin-8 |
| CA 15-3 (U/mL) | Circulating Cancer Antigen 15-3 |
| HGF (pg/mL) | Circulating Hepatocyte Growth Factor |
| OPG (ng/mL) | Circulating Osteoprotegerin |
| GDF15 (ng/mL) | Circulating Growth Differentiation Factor 15 |
| Leptin (pg/mL) | Circulating Leptin Concentration in pg/mL |
| Myeloperoxidase (ng/mL) | Circulating Myeloperoxidase |
| Kallikrein-6 (pg/mL) | Circulating Kallikrein-6 |
| TIMP-1 (pg/mL) | Circulating Tissue Inhibitor of Metalloproteinases 1 |
| Midkine (pg/mL) | Circulating Midkine |
| Prolactin (pg/mL) | Circulating Prolactin |
| Mesothelin (ng/mL) | Circulating Mesothelin |
| Galectin-3 (ng/mL) | Circulating Galectin-3 |
| OPN (pg/mL) | Circulating Osteopontin |
| NSE (ng/mL) | Circulating Neuron-Specific Enolase |
| sEGFR (pg/mL) | Circulating Soluble Epidermal Growth Factor Receptor |
| CEA (pg/mL) | Circulating Carcinoembryonic Antigen |
| AXL (pg/mL) | Circulating AXL Receptor Tyrosine Kinase |
| sPECAM-1 (pg/mL) | Circulating Soluble Platelet and Endothelial Cell Adhesion Molecule 1 |
| SHBG (nM) | Circulating Sex Hormone-Binding Globulin |
| Angiopoietin-2 (pg/mL) | Circulating Angiopoietin-2 |
| DKK1 (ng/mL) | Circulating Dickkopf WNT Signaling Pathway Inhibitor 1 |
| CYFRA 21-1 (pg/mL) | Circulating Cytokeratin-19 Fragment |
| PAR (pg/mL) | Circulating Protease-Activated Receptor |
| Endoglin (pg/mL) | Circulating Endoglin |
| FGF2 (pg/mL) | Circulating Fibroblast Growth Factor 2 |
| Follistatin (pg/mL) | Circulating Follistatin |

**Table S2:** Correlated Feature Pairs (*|Correlation|* > 0.5).

| First Feature | Second Feature | *\|Correlation\|* |
| --- | --- | --- |
| G-CSF (pg/mL) | IL-6 (pg/mL) | 0.778 |
| sHER2/sEGFR2/sErbB2 (pg/mL) | CYFRA 21-1 (pg/mL) | 0.769 |
| DKK1 (ng/mL) | NSE (ng/mL) | 0.517 |

**Table S3:** Correlated Mutual Information Analysis Results for Correlated Feature Pairs and Target Class.

| Dropped Feature | Selected Feature |
| --- | --- |
| G-CSF (0.33) | IL-6 (0.47) |
| sHER2/sEGFR2/sErbB2 (0.06) | CYFRA 21-1 (0.54) |
| DKK1 (0.00) | NSE (0.11) |

**Table S4:** Feature Reduction Across Multi-Stage Cancer Classification Levels**.**

| **Level** | **Removed Features** |
| --- | --- |
| 1^st^ | G-CSF, sHER2/sEGFR2/sErbB2, DKK1 |
| 2^nd^ | GDF15, IL-6, sHER2/sEGFR2/sErbB2 |
| 3^rd^ | HGF, Kallikrein-6, sHER2/sEGFR2/sErbB2 |
| 4^th^ | AXL, CD44, DKK1, GDF15, IL-6, Kallikrein-6, sEGFR, sPECAM-1, TIMP-2 |
| 5^th^ | CA-125, DKK1, Kallikrein-6, Midkine, Myeloperoxidase, sHER2/sEGFR2/sErbB2, sPECAM-1, TIMP-1 |
| 6^th^ | AXL, CD44, CYFRA 21-1, DKK1, HGF, IL-6, Kallikrein-6, sPECAM-1, TIMP-1, TIMP-2 |
| 7^th^ | CYFRA 21-1, DKK1, HGF, IL-6, Kallikrein-6, NSE, OPN, sHER2/sEGFR2/sErbB2 |

**Table S5:** Selected Feature Across Multi-Stage Cancer Classification Levels**.**

| **Level** | **Selected Features** | **Length** |
| --- | --- | --- |
| 1^st^ | IL-8, Ethnicity, IL-6, NSE, Omega Score, Age, OPN, Prolactin, TGFa, sEGFR, HE4, CYFRA 21-1, Thrombospondin-2, TIMP-1, TIMP-2, gender | 16 |
| 2^nd^ | sFas, CA 15-3, TGFa, CA19-9, HE4, IL-8, DKK1, Leptin, TIMP-2, CEA, ethonic, TIMP-1, Myeloperoxidase, CYFRA 21-1, NSE, Thrombospondin-2, Age, GCSF, Prolactin, gender, Omega Score | 21 |
| 3^rd^ | gender, TIMP-1, GDF15, CYFRA 21-1, ethonic, sFas, CA-125, Midkine, Thrombospondin-2, GCSF, TIMP-2, Follistatin, TGFa, OPG, Prolactin, IL-8, Mesothelin, Age, Omega Score | 19 |
| 4^th^ | ethonic, TGFa, AFP, Midkine, sFas, IL-8, Prolactin, CA 15-3, Thrombospondin-2, sHER2/sEGFR2/sErbB2, Leptin, GCSF, Myeloperoxidase, NSE, Age, gender, CYFRA 21-1, CA-125, TIMP-1, CA19-9 | 20 |
| 5^th^ | NSE, HGF, HE4, Galectin-3, sFas, CA19-9, IL-8, GDF15, TIMP-2, GCSF, gender, Thrombospondin-2, CD44, SHBG, AXL, OPN, ethonic, Omega Score, CYFRA 21-1, AFP, sEGFR | 21 |
| 6^th^ | sHER2/sEGFR2/sErbB2, GCSF, PAR, sFas, TGFa, OPG, CEA, Omega Score, AFP, CA19-9, Thrombospondin-2, HE4, Myeloperoxidase, Age, CA-125, ethonic, gender, Midkine | 18 |
| 7^th^ | sFas, gender, FGF2, AXL, IL-8, CEA, sEGFR, CA-125, CA 15-3, GDF15, Galectin-3, Omega Score, HE4, Follistatin, TIMP-2, CA19-9, PAR, Prolactin, sPECAM-1, GCSF, TIMP-1, Thrombospondin-2, TGFa, Leptin, ethonic, Myeloperoxidase | 26 |

**Table S6:** Best Hyperparmeters Across Multi-Stage Cancer Classification Levels**.**

| **Level** | **n_estimators** | **num_leaves** | **learning_rate** | **Balanced Accuracy** |
| --- | --- | --- | --- | --- |
| 1^st^ | 150 | 500 | 0.21 | 98.57% |
| 2^nd^ | 200 | 398 | 0.54 | 82.02% |
| 3^rd^ | 250 | 439 | 0.87 | 86.09% |
| 4^th^ | 50 | 106 | 0.42 | 80.13% |
| 5^th^ | 150 | 500 | 0.21 | 85.34% |
| 6^th^ | 50 | 106 | 0.42 | 94.12% |
| 7^th^ | 200 | 398 | 0.54 | 96.21% |

**Table S7:** Performance Results Across Multi-Stage Cancer Classification Levels**.**

| **Level** | **Precision** | **Recall** | **F1-score** | **Accuracy** | **AUC** |
| --- | --- | --- | --- | --- | --- |
| 1^st^ | 100% | 99.01% | 99.5% | 99.45% | 99.95% |
| 2^nd^ | 91.23% | 83.87% | 87.39% | 85.15% | 92.51% |
| 3^rd^ | 90.91% | 97.56% | 94.12% | 91.94% | 98.37% |
| 4^th^ | 90% | 81.82% | 85.71% | 92.68% | 94.85% |
| 5^th^ | 90.48% | 100% | 95% | 93.33% | 100% |
| 6^th^ | 90.91% | 100% | 95.24% | 95% | 99% |
| 7^th^ | 100% | 100% | 100% | 100% | 100% |

**Table S8:** Selected Features using Different Top Feature Subset Sizes.

| **#top features** | **Features** |
| --- | --- |
| 5 | IL-8, Eethonic, NSE, IL-6, Age, OPN, Prolactin, Omega Score, CYFRA 21-1 |
| 8 | IL-8, Ethonic, NSE, IL-6, Age, Omega Score, OPN, Prolactin, Thrombospondin-2, CYFRA 21-1, TIMP-2, gender |
| 10 | IL-8, Ethonic, NSE, IL-6, Age, Omega Score, OPN, Prolactin, sEGFR, TGFa, HE4, Thrombospondin-2, CYFRA211, TIMP-1, TIMP-2, gender |
| 12 | IL-8, Ethonic, NSE, IL-6, Age, Omega Score, OPN, Prolactin, sEGFR, TGFa, HE4, Myeloperoxidase, CYFRA 21-1, Thrombospondin-2, OPG, TIMP-2, TIMP-1, sFas, gender |
| 15 | IL-8, Ethonic, NSE, IL-6, Age, Omega Score, OPN, Prolactin, sEGFR, TGFa, HE4, Myeloperoxidase, HGF, CYFRA 21-1, sFas, PAR, Thrombospondin-2, OPG, TIMP-2, Leptin, TIMP-1, Midkine, SHBG, sPECAM-1, gender |

**Table S9:** Confusion Matrix for Cancer Detection on Hinestrosa et al. Dataset.

|  | | **Predicted** | |
| --- | --- | --- | --- |
|  |  | Cancer | Normal |
| **Actual** | Cancer | 128 (TP) | 54 (FN) |
|  | Normal | 43 (FP) | 325 (TN) |
